# Синдромальная задержка роста, обусловленная нарушением функции рибосомального белка eL13

**DOI:** 10.14341/probl13377

**Published:** 2023-10-23

**Authors:** Н. А. Макрецкая, И. Г. Воронцова, А. А. Буянова, Д. О. Коростин, Е. Е. Петряйкина, А. Н. Тюльпаков

**Affiliations:** Медико-генетический научный центр имени академика Н.П. Бочкова; Российская детская клиническая больница; Центр высокоточного редактирования и генетических технологий для биомедицины ФГАОУ ВО РНИМУ им. Н.И. Пирогова Минздрава России; Центр высокоточного редактирования и генетических технологий для биомедицины ФГАОУ ВО РНИМУ им. Н.И. Пирогова Минздрава России; Российская детская клиническая больница; Медико-генетический научный центр имени академика Н.П. Бочкова; Российская детская клиническая больница

**Keywords:** низкорослость, скелетная дисплазия, спондилоэпиметафизарная дисплазия, RPL13

## Abstract

Задержка роста более 2 SD ниже среднего популяционного или предполагаемого семейного целевого роста классифицируется как низкорослость и может быть клиническим проявлением большого числа заболеваний. Использование в последние годы новейших методик молекулярно-генетического анализа позволило лучше понять патогенез наследственных форм низкорослости. Одними из недавно открытых механизмов развития данной патологии являются гетерозиготные патогенные варианты в гене RPL13, ассоциированные со спондилоэпиметафизарной дисплазией (СЭД) тип Исидора-Туте. Характерными фенотипическими особенностями для данной формы являются нормальные показатели роста при рождении, выраженная постнатальная задержка роста, платиспондилия, эпифизарные дефекты проксимального отдела бедренной кости, coxa vara, genu varum. В настоящем исследовании представлены клинико-рентгенологические характеристики первого в Российской Федерации пациента со СЭД, вызванной мутацией в гене RPL13.

## Abstract (en)

Growth retardation for more than 2 SD below the average population or presumed familial target height is classified as a short stature and may be a clinical manifestation of a large number of disorders. The use of the latest methods of molecular genetic analysis in recent years has allowed for a better understanding of the pathogenesis of inherited forms of a short stature. One of the recently discovered mechanisms of this pathology was monoallelic mutations in RPL13 gene, leading to the development of Isidor-Toutain type spondyloepimetaphyseal dysplasia (SEDM). Characteristic phenotypic features for this form are normal birth length, early postnatal growth deficiency, platyspondyly, proximal femoral epiphyseal changes, coxa vara, genu varum. This study presents the clinical and radiological characteristics of the first patient in the Russian ­Federation with SEMD caused by a mutation in RPL13 gene.

## АКТУАЛЬНОСТЬ

Низкорослость — это статистическое определение, представляющее собой отставание роста на два и более стандартных отклонения (SD) от среднего значения показателя для конкретной группы населения с поправкой на пол и возраст. Нарушение показателей роста и/или его скорости являются одним из основных поводов обращения к педиатру или эндокринологу. Несмотря на то, что в ряде случаев причины нарушения роста могут быть легко идентифицированы при сборе анамнеза, физикальном осмотре, биохимических и гормональных исследованиях, как, например, при целиакии, СТГ-дефиците, гипотиреозе, заболеваниях кишечника и других, в большинстве случаев патогенез и, главное, молекулярная основа развития данного состояния остается неясна. Однако установка этиологической причины низкорослости является основополагающим фактором при выборе тактики лечения и наблюдения.

С патофизиологической точки зрения наследственные варианты низкорослости можно разделить на следующие подгруппы: дефекты, приводящие к нарушению функции оси гормон роста — ИФР-1; дефекты, приводящие к нарушению внутриклеточных сигнальных путей или фундаментальных клеточных процессов; и дефекты, приводящие к нарушению паракринной регуляции или активации транскрипционных факторов, присутствующих в ростовой пластине и необходимых для ее правильного развития [1]. К последней относятся наследственные скелетные дисплазии, в частности — спондилоэпиметафизарные дисплазии (СЭД).

Отличительной особенностью СЭД является сочетанное поражение тел позвонков и эпифизов трубчатых костей, и, как следствие, задержка роста с нарушением пропорций тела, которые могут быть клинически не выражены, особенно у детей раннего возраста. Неспецифичность и вариабельность клинических проявлений при данной патологии усложняют проведение дифференциальной диагностики как среди СЭД, так и с вариантами низкорослости, обусловленными эндокринными нарушениями.

Внедрение новых методов молекулярно-генетического исследования, в том числе высокопроизводительного секвенирования, позволило расширить наши представления о молекулярной основе наследственных заболеваний и вследствие этого улучшить диагностику данных состояний. Одним из заболеваний, для которого сравнительно недавно была установлена моногенная этиология, является спондилоэпиметафизарная дисплазия тип Исидора-Туте (Isidor-Toutain) [2]. В 2019 г. Le Caignec с соавт. показали, что данная форма СЭД обусловлена нарушениями в гене RPL13 [3], и к настоящему моменту в мировой литературе описаны лишь единичные случаи данного заболевания [2–5].

В данной работе впервые в Российской Федерации представлены клинические, лабораторные и рентгенологические данные пациента с задержкой роста, обусловленной мутацией в гене RPL13.

## ОПИСАНИЕ СЛУЧАЯ

Девочка от второй беременности (первая беременность — мальчик, здоров), протекавшей на фоне анемии легкой степени тяжести, в 12 недель — острый бронхит, в 23 недели — острый тубулоинтестинальный нефрит, острый пиелонефрит, в 30 недель — хронический пиелонефрит. При проведении ультразвуковой диагностики на сроке в 31 неделю выявлено укорочение трубчатых костей. Роды срочные, самостоятельные на 38-й неделе гестации. Длина при рождении — 42 см (-3.4 SD), масса — 2730 г (-0.9 SD). В первые сутки жизни у ребенка развилась дыхательная недостаточность, потребовавшая перевода на ИВЛ. В возрасте 1 мес проведена трахеостомия. В возрасте 1,5 года — попытка проведения деканюлизации, в динамике нарастающая дыхательная недостаточность, повторный перевод на ИВЛ.

В возрасте 3 лет 10 мес пациентка обследована эндокринологом в связи с наличием прогрессирующей задержки роста, диспропорций тела. При осмотре: рост — 69,3 см (-7.05 SD), верхний сегмент — 45,3 см (-5.4 SD), нижний сегмент — 24,0 см (-7.0 SD), вес — 10 кг (ИМТ SDS +2.8), окружность головы — 49 см (-0,61 SD). При осмотре обращали на себя внимание мелкие черты лица, преобладание мозгового черепа над лицевым, голубой оттенок склер, деформация грудной клетки, коленных суставов, кифоз, отмечалось ограничение подвижности тазобедренных суставов. Рост матери —153 см, отца — 174 см.

В биохимическом анализе крови клинически значимых изменений нет: кальций общий — 2,49 ммоль/л, кальций ионизированный — 1,28 ммоль/л, фосфор неорганический — 1,89 ммоль/л. В гормональном профиле уровень ИФР-1 соответствует возрастной норме — 81,7 нг/л (18,2–172,0). По данным рентгенографии нижних конечностей, в прямой проекции выявлена выраженная варусная деформация коленных суставов. Метаэпифизы всех костей, образующих правый и левый коленные суставы, расширены, вздуты. Соотношения в суставах неправильные (больше — в правом суставе). Суставные поверхности неровные, нечеткие, суставные щели неравномерные, расширены (рис. 1–2).

**Figure:**
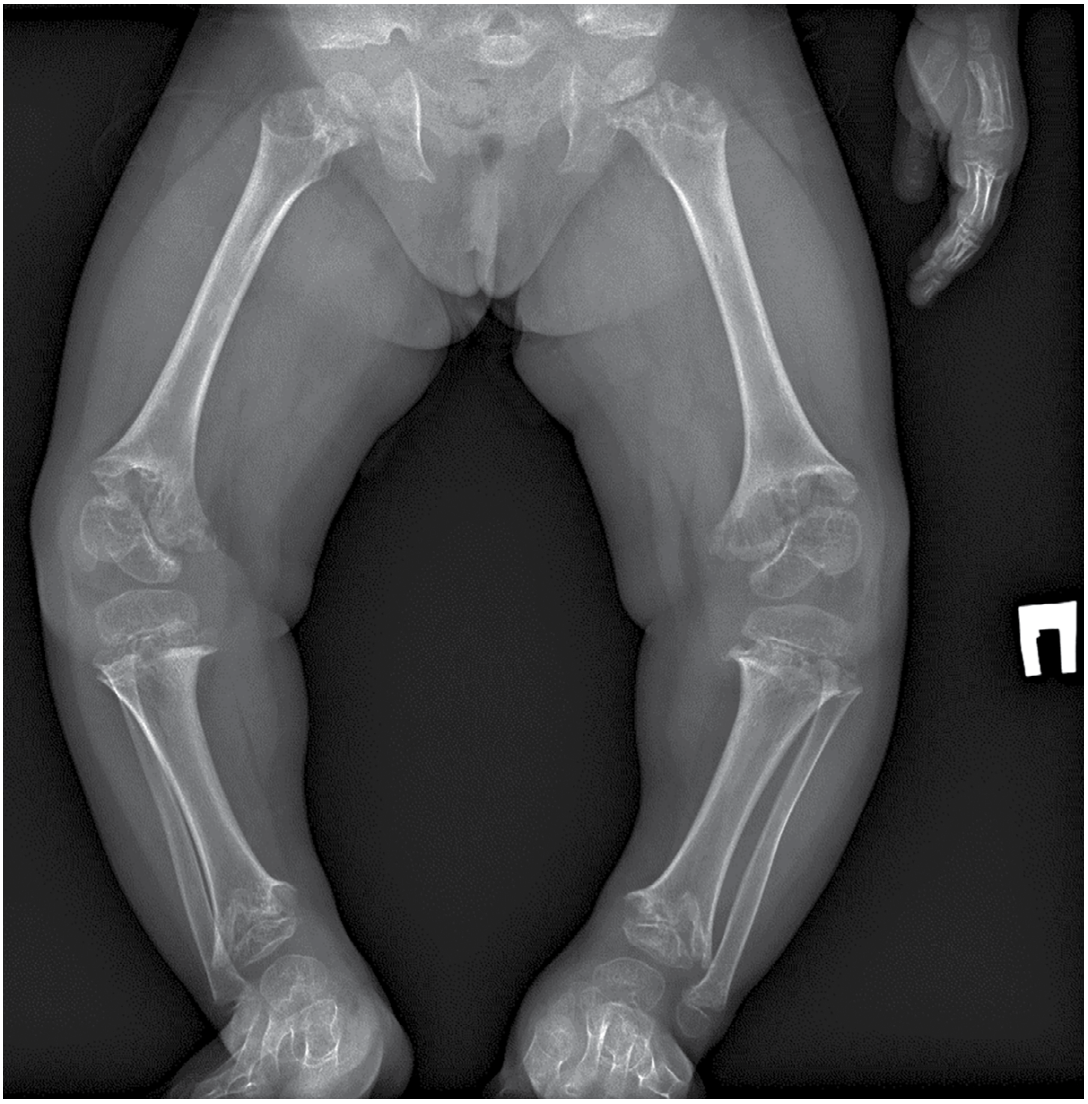
Рисунок 1. Рентгенография коленного и тазобедренного суставов в прямой проекции.

**Figure:**
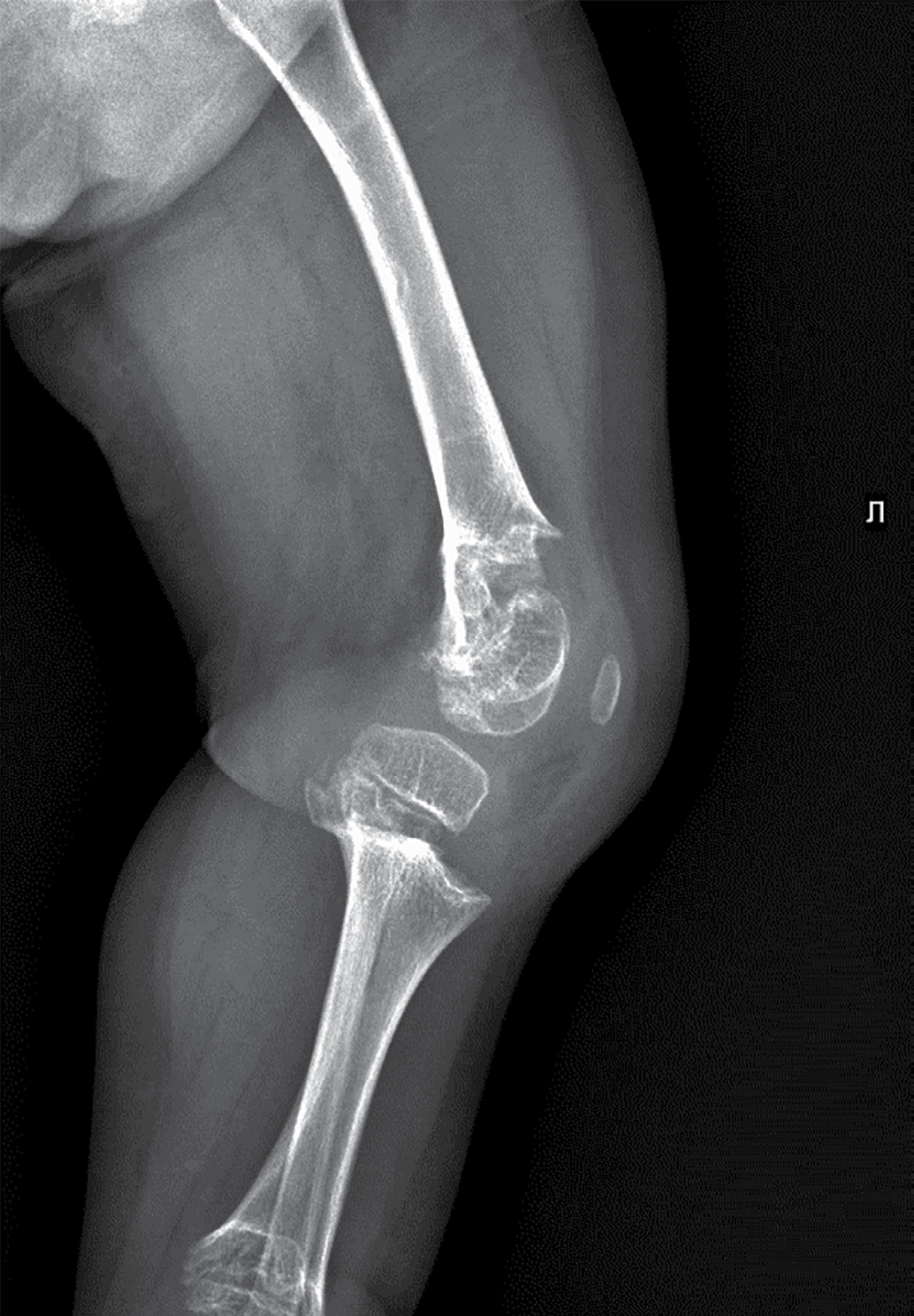
Рисунок 2. Рентгенография правого коленного сустава в боковой проекции.

Выявлен ранее не описанный вариант нуклеотидной последовательности в гетерозиготном состоянии в экзоне 6 гена RPL13 (NM_000977.4: c.499C>T), приводящий к появлению сайта преждевременной терминации трансляции в кодоне 167 (p.Arg167Ter). Указанный вариант нуклеотидной последовательности отсутствует в базе популяционных частот gnomAD. При обследовании родителей пациента изменений в указанном фрагменте гена RPL13 не выявлено, что позволяет сделать вывод о происхождении выявленного у пробанда варианта de novo. В соответствии с критериями, используемыми для интерпретации результатов высокопроизводительного секвенирования [7][8], вариант оценен как патогенный (PM2, PVS1, PS2).

## ОБСУЖДЕНИЕ

Isidor с соавт. в 2013 г. опубликована клиническая характеристика нового варианта СЭД у двух неродственных сибсов [2]. Основными фенотипическими особенностями являлись нормальная длина тела при рождении; снижение скорости роста в раннем постнатальном периоде; низкорослость; сгибательные контрактуры в тазобедренном суставе; genu varum; спондилоэпиметафизарные аномалии: платиспондилия, тяжелые прогрессивные метафизарные изменения, уменьшение размеров эпифизов проксимального отдела бедренной кости с выраженным coxa vara, метафизарные аномалии верхних конечностей легкой степени и отсутствие кальцификатов; отсутствие нарушений интеллектуального развития и дополнительных скелетных аномалий.

Ген RPL13 картирован на 16 хромосоме в локусе 16q24.3, кодирует рибосомальный белок eL13, состоящий из 211 аминокислотных остатков и являющийся компонентом рибосомальной субъединицы 60S человека [9]. В работе Le Caignec продемонстрировано увеличение количества предшественников 45S и 41S пре-рРНК в нокаутных по PRL13 клетках HeLa, что приводило к раннему дефекту процессинга пре-рРНК [3]. Однако не все описанные в данной работе варианты оказывали значимый вклад в биосинтез рибосом, в связи с чем авторы предполагают, что PRL13 может играть более значимую роль в трансляции определенных мРНК в хондроцитах и/или остеобластах.

Заболевание крайне редкое: в настоящий момент в мировой литературе описано 25 пациентов из 15 семей с СЭД, обусловленной различными мутациями в гене RPL13 [2–5]. Тип наследования заболевания — аутосомно-доминантный. Интересно, что во всех случаях заболевание было обусловлено мутациями в двух горячих точках — сайте сплайсинга интрона 5 (60%) и 6 экзоне, в котором обнаружили миссенс-варианты (40%). Кроме того, для данного варианта СЭД более характерны мутации de novo (67%). Систематический анализ клинических и рентгенологических особенностей позволил установить основные характеристики данной формы СЭД [4]:

Несмотря на то, что задержка роста выделена как одна из фенотипических особенностей для СЭД, обусловленной мутациями в гене RPL13, описана неполная пенетрантность данного признака даже внутри одной семьи, и ряд пациентов имели нормальные показатели роста [4][5].

У нашей пациентки диагностированы ранее не описанные фенотипические особенности, в частности наличие внутриутробной задержки роста и развитие дыхательной недостаточности сразу после рождения. Не исключено, что данные состояния связаны с сопутствующими заболеваниями во время беременности и развитием фетоплацентарной недостаточности, а не с нарушением функции белка eL13. Между тем ген RPL13 экспрессирован повсеместно, и можно ожидать, что у пациентов могут выявляться внескелетные изменения. На это может указывать, например, публикация Díaz-González с соавт., которые описали в своей когорте у пациентов колобому радужки, миопию и гипермобильность суставов [4]. Следует также отметить, что, в отличие от всех ранее описанных вариантов в RPL13, выявленная нами нуклеотидная замена должна приводить к укорочению белка, что может специфически влиять на его функцию.

## Заключение

Впервые в отечественной литературе описан клинический случай спондилоэпифизарной дисплазии, обусловленной патогенным вариантом в гене RPL13. Представленное наблюдение подчеркивает сложность дифференциальной диагностики низкорослости у детей раннего возраста. Проведение полноэкзомного секвенирования в таких случаях является важным диагностическим методом для определения молекулярной природы заболевания и решения вопроса о дальнейшей тактике ведения пациента.

## Дополнительная информация

Источники финансирования. Работа выполнена в рамках государственного задания Минобрнауки России для ФГБНУ «МГНЦ».

Конфликт интересов. Авторы декларируют отсутствие явных и потенциальных конфликтов интересов, связанных с содержанием настоящей статьи.

Участие авторов. Макрецкая Н.А. — существенный вклад в дизайн исследования, сбор материала, анализ полученных данных, написание текста; Воронцова И.Г. — сбор материала, анализ полученных данных, редактирование текста; Буянова А.А. — проведение аналитической части молекулярно-генетического исследования, редактирование текста; Коростин Д.О. — проведение молекулярно-генетического исследования; Петряйкина Е.Е. — сбор материала, анализ полученных данных, дизайн исследования, редактирование текста; Тюльпаков А.Н. — концепция и дизайн исследования, внесение в рукопись существенной правки с целью повышения научной ценности статьи.

Все авторы одобрили финальную версию статьи перед публикацией, выразили согласие нести ответственность за все аспекты работы, подразумевающую надлежащее изучение и решение вопросов, связанных с точностью или добросовестностью любой части работы.

Согласие пациента. Добровольное информированное согласие законных представителей пациента на публикацию в журнале «Проблемы эндокринологии» получены.
